# Prognostic factors for different outcomes in patients with metastatic spinal cord compression from cancer of unknown primary

**DOI:** 10.1186/1471-2407-12-261

**Published:** 2012-06-21

**Authors:** Sarah Douglas, Stefan Huttenlocher, Amira Bajrovic, Volker Rudat, Steven E Schild, Dirk Rades

**Affiliations:** 1 Department of Radiation Oncology, University of Lubeck, Lubeck, Germany; 2 Department of Radiation Oncology, University Medical Center Hamburg-Eppendorf, Hamburg, Germany; 3 Department of Radiation Oncology, Saad Specialist Hospital Al-Khobar, Al-Khobar, Saudi Arabia; 4 Department of Radiation Oncology, Mayo Clinic Scottsdale, Scottsdale, AZ, USA

**Keywords:** Prognostic factors, Cancer of unknown primary, Metastatic spinal cord compression, Radiotherapy, treatment outcomes

## Abstract

**Background:**

Patients with cancer of unknown primary account for 10% of patients with metastatic spinal cord compression (MSCC). This retrospective study was performed to identify prognostic factors for functional outcome, local control of MSCC, and survival in 175 of such patients treated with radiotherapy alone.

**Methods:**

Investigated were nine potential prognostic factors including age, gender, Eastern Cooperative Oncology Group performance score (ECOG-PS), number of involved vertebrae, pre-radiotherapy ambulatory status, other bone metastases, visceral metastases, time developing motor deficits before radiotherapy, and the radiation schedule.

**Results:**

On multivariate analysis, better functional outcome was associated with absence of visceral metastases (estimate: 0.72; 95%-confidence interval [CI]: 0.07-1.36; p = 0.030) and a slower (>7 days) development of motor deficits (estimate: 1.93; 95%-CI: 1.18-2.68; p < 0.001). Improved local control of MSCC was associated with absence of visceral metastases (risk ratio [RR]: 10.26; 95%-CI: 2.11-74.73; p = 0.004). Improved survival was associated with favorable ECOG-PS (RR: 2.12; 95%-CI: 1.40-3.29; p < 0.001), being ambulatory prior to radiotherapy (RR: 1.98; 95%-CI: 1.40-2.81; p < 0.001), absence of visceral metastases (RR: 2.74; 95%-CI: 1.93-3.91; p < 0.001), and slower development of motor deficits (RR: 1.27; 95%-CI: 1.07-1.51; p = 0.007). Absence of other bone metastases showed a trend (RR: 1.38; 95%-CI: 0.98-1.95; p = 0.07).

**Conclusions:**

This study identified additional independent prognostic factors for functional outcome, local control of MSCC, and survival after radiotherapy of MSCC from cancer of unknown primary. These prognostic factors can help select the best treatment regimen for each individual patient.

## Background

Up to 10% of adult cancer patients develop metastatic spinal cord compression (MSCC) during their disease [1,2]. Today the most common treatment modality used for MSCC is radiotherapy alone. The addition of upfront decompressive surgery to radiotherapy can improve the outcomes of those 10-15% of patients with MSCC, who have a favorable ECOG-PS, a relatively good survival prognosis, and a limited number of involved vertebrae [2,3]. To optimally take into account each patient’s individual situation, it is mandatory to personalize the treatment approach as much as possible. Personalization of treatment can be facilitated if patients with MSCC from a particular type of primary tumor are regarded as a separate group of patients. Such an approach appears reasonable, because tumor entities can vary a lot regarding the prognosis of the disease and the biological behavior. Patients with MSCC from cancer of unknown primary demand particular attention, because they have a very poor survival prognosis when compared to patients with MSCC from other tumors [1,2]. Furthermore, these patients are relatively common and account for about 10% of all patients developing MSCC. This study aimed to identify independent prognostic factors for different endpoints including functional outcome, local control of MSCC, and survival in the largest series of patients with MSCC from cancer of unknown primary reported so far, in order to contribute to the personalization of the treatment of these patients.

## Results

Of the 175 patients included in this study, 21 (12%) showed an improvement, 96 (55%) no further progression (no change), and 58 (33%) deterioration of motor function (21% real deterioration plus 12% no improvement of complete paraplegia). Summarized in Table 1 are the results of the multivariate analysis of functional outcome. An improvement of motor function was significantly associated with absence of visceral metastases at the time of radiotherapy (estimate: 0.72; 95%-confidence interval [CI]: 0.07-1.36; p = 0.030) and slower (>7 days) development of motor deficits prior to the start of radiotherapy (estimate: 1.93; 95%-CI: 1.18-2.68; p < 0.001).

**Table 1:** Impact of the potential prognostic factors on functional outcome

	**Improvementn (%)**	**No changen (%)**	**Deterioration n (%)**	**p**
Age				
≤65 years (n = 85)	**9 (11)**	53 (62)	**23 (27)**	
**>65 years (n = 90)**	**12 (13)**	**43 (48)**	**35 (39)**	**0.54**
Gender				
**Female (n = 53)**	**8 (15)**	**27 (51)**	**18 (34)**	
**Male (n = 122)**	**13 (11)**	**69 (57)**	**40 (33)**	**0.42**
ECOG performance score				
**2 (n = 49)**	**10 (20)**	**29 (59)**	**10 (20)**	
**3-4 (n = 126)**	**11 (9)**	**67 (53)**	**48 (38)**	**0.25**
Number of involved vertebrae				
**1-2 (n = 55)**	**8 (15)**	**35 (64)**	**12 (22)**	
**≥3 (n = 120)**	**13 (11)**	**61 (51)**	**46 (38)**	**0.84**
Ambulatory status prior to RT				
**Not Ambulatory (n = 89)**	**9 (10)**	**46 (52)**	**34 (38)**	
**Ambulatory (n = 86)**	**12 (14)**	**50 (58)**	**24 (28)**	**0.21**
Other bone metastases				
**No (n = 65)**	**11 (17)**	**39 (60)**	**15 (23)**	
**Yes (n = 110)**	**10 (9)**	**57 (52)**	**43 (39)**	**0.46**
Visceral metastases				
**No (n = 86)**	**15 (17)**	**34 (40)**	**37 (43)**	
**Yes (n = 89)**	**6 (7)**	**62 (70)**	**21 (24)**	0.030
Time developing motor deficits				
**1-7 days (n = 88)**	**4 (5)**	**38 (43)**	**46 (52)**	
**>7 days (n = 87)**	**17 (20)**	**58 (67)**	**12 (14)**	<0.001
Radiation schedule				
**Short-course RT (n = 90)**	**7 (8)**	**60 (67)**	**23 (26)**	
**Longer-course RT (n = 85)**	**14 (16)**	**36 (42)**	**35 (41)**	**0.86**
Entire cohort (n = 175)	**21 (12)**	**96 (55)**	**58 (33)**	

The p-values were obtained from the multivariate analysis performed with the ordered-logit model.

An improvement of local control of MSCC was associated with the absence of visceral metastases at the time of radiotherapy (p = 0.001) in the univariate analysis. The results of the univariate analysis are summarized in Table 2. On multivariate analysis, improved local control was also significantly associated with absence of visceral metastases (risk ratio [RR]: 10.26; 95%-CI: 2.11-74.73; p = 0.004).

**Table 2:** Univariate analysis of local control of MSCC

	**At 6 months (%)**	**At 12 months (%)**	**p**
Age			
**≤65 years (n = 85)**	**95**	**95**	
**>65 years (n = 90)**	**92**	**61**	**0.13**
Gender			
**Female (n = 53)**	**96**	**96**	
**Male (n = 122)**	**92**	**75**	**0.35**
ECOG performance score			
**2 (n = 49)**	**98**	**83**	
**3-4 (n = 126)**	**88**	**88**	**0.44**
Number of involved vertebrae			
**1-2 (n = 55)**	**98**	**82**	
**≥3 (n = 120)**	**90**	**82**	**0.27**
Ambulatory status prior to RT			
**Not Ambulatory (n = 89)**	**92**	**92**	
**Ambulatory (n = 86)**	**93**	**80**	**0.54**
Other bone metastases			
**No (n = 65)**	**98**	**79**	
**Yes (n = 110)**	**88**	**88**	**0.55**
Visceral metastases			
**No (n = 86)**	**98**	**91**	
**Yes (n = 89)**	**86**	**43**	0.002
Time developing motor deficits			
**1-7 days (n = 88)**	**89**	**89**	
**>7 days (n = 87)**	**96**	**83**	**0.22**
Radiation schedule			
**Short-course RT (n = 90)**	**87**	**77**	
**Longer-course RT (n = 85)**	**98**	**86**	**0.12**
Entire cohort (n = 175)	**93**	**82**	

**Figure 1:**
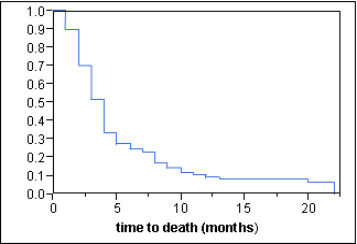
Overall survival of the entire cohort (Kaplan-Meier curve).

The median survival time was 4 months in the entire cohort (Figure 1). According to the univariate analysis of survival, improvement was associated with age <65 years (p = 0.048), an ECOG-PS 2 (p < 0.001), being ambulatory prior to radiotherapy (p < 0.001), absence of other bone metastases at the time of radiotherapy (p = 0.005), absence of visceral metastases at the time of radiotherapy (p < 0.001), and slower development of motor deficits prior to radiotherapy (p < 0.001). The results of the univariate analysis of survival are summarized in Table 3. The multivariate analysis revealed four prognostic factors to be associated with improvement of survival: ECOG-PS 2 (RR: 2.12, 95%-CI: 1.40–3.29, p < 0.001), being ambulatory (RR: 1.98, 95%-CI: 1.40–2.81, p < 0.001), the absence of visceral metastases (RR: 2.74, 95%-CI: 1.93–3.91, p < 0.001) and the time developing motor deficits (RR: 1.27, 95%-CI: 1.07–1.51, p = 0.007). Absence of other bone metastases showed a trend (RR: 1.38; 95%-CI: 0.98-1.95; p = 0.07). The results of the multivariate analysis of survival are shown in Table 4.

**Table 3:** Univariate analysis of survival

	**At 6 months (%)**	**At 12 months (%)**	**p**
Age			
**≤65 years (n = 85)**	**32**	**8**	
**>65 years (n = 90)**	**19**	**12**	0.048
Gender			
**Female (n = 53)**	**26**	**13**	
**Male (n = 122)**	**25**	**8**	**0.38**
ECOG performance score			
**2 (n = 49)**	**53**	**24**	
**3-4 (n = 126)**	**14**	**4**	<0.001
Number of involved vertebrae			
**1-2 (n = 55)**	**33**	**9**	
**≥3 (n = 120)**	**22**	**10**	**0.13**
Ambulatory status prior to RT			
**Not Ambulatory (n = 89)**	**15**	**3**	
**Ambulatory (n = 86)**	**36**	**17**	<0.001
Other bone metastases			
**No (n = 65)**	**38**	**15**	
**Yes (n = 110)**	**17**	**7**	0.005
Visceral metastases			
**No (n = 86)**	**45**	**16**	
**Yes (n = 89)**	**6**	**3**	<0.001
Time developing motor deficits			
**1-7 days (n = 88)**	**10**	**1**	
**>7 days (n = 87)**	**40**	**18**	<0.001
Radiation schedule			
**Short-course RT (n = 90)**	**21**	**11**	
**Longer-course RT (n = 85)**	**29**	**8**	**0.19**
Entire cohort (n = 175)	**25**	**9**	

**Table 4:** Multivariate analysis of survival (Cox proportional hazards model)

	**Risk ratio**	**95%- confidence interval**	**p**
Age			
**(≤65*vs.*>65 years)**	**1.14**	**0.83 – 1.58**	**0.42**
ECOG performance score			
**(2*vs.*3–4)**	**2.12**	**1.40 – 3.29**	<0.001
Ambulatory status prior to RT			
**(ambulatory*vs.*ot ambulatory)**	**1.98**	**1.40 – 2.81**	<0.001
Other bone metastases			
**(no*vs.*yes)**	**1.38**	**0.98 – 1.95**	**0.07**
Visceral metastases			
**(no*vs.*yes)**	**2.74**	**1.93 – 3.91**	<0.001
Time developing motor deficits			
**(>7*vs.*1–7 days**	**1.27**	**1.07 – 1.51**	0.007

Acute radiation induced toxicity such as skin toxicity, nausea and diarrhea was mild, late toxicity such as myelopathy was not observed.

## Discussion

The treatment for patients with MSCC should be planned by taking into account independent prognostic factors, which allow estimating the patient’s prognosis. Prognostic factors that indicate the effect of radiotherapy on functional outcome are important to identify patients who appear adequately treated with radiotherapy alone, *i.e.* patients who have a high probability to maintain or regain the ability to walk after irradiation. Such prognostic factors can also identify patients who do not achieve a satisfying functional outcome with radiotherapy alone and, therefore, could benefit from upfront decompressive surgery in addition to radiotherapy. In randomized study of 101 highly selected patients that compared decompressive surgery followed by radiotherapy to radiotherapy alone, significantly more patients were able to walk after treatment in the surgery plus radiotherapy group (84% *vs.* 57%, p = 0.001) [3].

To be able to predict the patient’s survival prognosis is also very important for the selection of the appropriate treatment regimen. Patients with a more favorable prognosis are likely to benefit from longer-course radiotherapy programs supplemented by bisphosphonate treatment rather than from short-course radiotherapy. This is because short-course radiotherapy results in worse local control of MSCC, which becomes more of an issue in patients surviving 6 months or longer following treatment [4,5]. Prognostic factors that allow predict survival are important also for identifying patients with a very poor survival prognosis, for whom a short-course radiotherapy would be a better option in order to avoid unnecessary distress for these often debilitated patients.

This study aimed to identify prognostic factors for both functional outcome and survival, because both endpoints are important in order to choose the most appropriate treatment regimen for the individual patient with MSCC from cancer of unknown primary. The multivariate analysis of functional outcome revealed that patients who had visceral metastases at the time of radiotherapy and experienced a rapid development of motor deficits prior to radiotherapy had an unfavorable functional outcome. These findings agree with our previous report on MSCC from cancer of unknown primary published five years ago [6]. The finding that a rapid development of motor deficits was associated with a worse functional outcome could be explained by the fact that a rapid decline in motor function was caused by disruption of the arterial blood flow resulting in spinal cord infarction [7,8]. In contrast, a slower decline in motor function was most likely a result of venous congestion, which was reversible in many cases. In our present study, 21% of the patients showed a deterioration of motor function after radiotherapy alone, and 12% did not improve after complete paraplegia. In a recent retrospective study of 51 patients who received surgical management of MSCC from CUP, deterioration was observed only in 6% of patients [9]. Therefore, it appears that a considerable proportion of patients with MSCC from cancer of unknown primary may be considered for decompressive surgery, in particular those patients with a rapid development of motor deficits or visceral metastases who do not have an extraordinarily poor survival prognosis.

Prognostic factors predicting the patient’s survival prognosis are important in two ways. They can help identify patients who may not be candidates for decompressive surgery because they have a very poor survival prognosis, although functional outcome following radiotherapy alone is not expected to be satisfying. Furthermore, the estimated survival time has an impact on the selection of the radiotherapy regimen. In the present study, survival was negatively associated with four prognostic factors indicating an advanced and rapidly progressing disease: poor performance status, being not ambulatory prior to radiotherapy, presence of visceral metastases, and rapid development of motor deficits prior to radiotherapy. Patients with these negative predictors may receive short-course radiotherapy to avoid that these patients have to spend a considerable part of their remaining life time with treatment. In the group of patients (n = 28) with all four negative prognostic factors, the 6-month survival rate was only 4%. These patients may be candidates for single-fraction radiotherapy or best supportive care.

The prognostic value of the ECOG-PS has not been observed in our previous study published five years ago and can, therefore, be considered a new prognostic factor for patients with MSCC from cancer of unknown primary [6]. Potential prognostic factors for local control of MSCC have not yet been investigated in patients with MSCC from cancer of unknown primary at all. Therefore, the present study provides new and important results in addition to our previous report. However, the retrospective nature of this study must be taken into account during the interpretation of the results. Retrospective studies always bear the risk of hidden selection biases. However, a prospective study will be difficult to perform in patients with MSCC from cancer of unknown primary, as it will take several years to include a sufficiently large number of such patients.

## Conclusions

The present study identified independent prognostic factors for functional outcome, local control of MSCC, and survival in patients with MSCC from cancer of unknown primary. These prognostic factors can guide physicians to decide which is the most appropriate treatment regimen for the individual patient. During such a decision making process the questions whether decompressive surgery may be a reasonable option and whether short-course or longer-course radiotherapy is more appropriate should be adressed.

## Methods

The data of 175 patients irradiated for MSCC from cancer of unknown primary between 1991 and 2011 were retrospectively reviewed. The patients had to fulfill the following criteria to be included in this analysis: motor deficits of the lower extremities caused by metastatic compression of the thoracic or lumbar spinal cord, confirmation of the diagnosis with spinal computed tomography or magnet resonance imaging, and no previous surgery or radiotherapy within the involved parts of the spinal cord. The patients were given a daily dexamethasone dose between 3x4 mg and 4x8 mg from the first day of radiotherapy and then at least for another week. The patients were generally presented to a neurosurgeon or an orthopedic surgeon to discuss whether upfront decompressive surgery may be a reasonable option or not. Patients were generally not considered for surgery if they had a poor performance status (ECOG 3–4), involvement of several spinal sites, severe neurologic deficits (paraplegia) for longer than 48 hours, and a poor expected survival in case of multiple extraosseous lesions. Only 10% of the patients who presented with MSCC from CUP were considered candidates for surgery. The data were collected from the patients themselves, their files, and their general practitioners or treating oncologists. Because this study did not report on a clinical trial, and because the data were retrospective in nature and analyzed anonymously, approval by an ethic committee was not necessary. The patient characteristics are summarized in Table 5.

**Table 5:** Patient characteristics

	**N patients**	**Proportion (%)**
Age		
**≤65 years**	**85**	**49**
**>65 years**	**90**	**51**
Gender		
**Female**	**53**	**30**
**Male**	**122**	**70**
ECOG performance score		
**2**	**49**	**78**
**3-4**	**126**	**72**
Number of involved vertebrae		
**1-2**	**55**	**31**
**≥3**	**120**	**69**
Ambulatory status prior to RT		
**Not Ambulatory**	**89**	**51**
** Ambulatory**	**86**	**49**
Other bone metastases		
**No**	**65**	**37**
**Yes**	**110**	**63**
Visceral metastases		
**No**	**86**	**49**
**Yes**	**89**	**51**
Time developing motor deficits		
1-7 days	88	50
**>7 days**	**87**	**50**
**Radiation schedule**		
Short-course RT	90	51
**Longer-course RT**	**85**	**49**

Every participating center contributed an unselected series of patients with MSCC treated within a specific time period. The energy used for irradiation varied between 6–10 MeV photons, and the planning target volume included one uninvolved vertebra above and below the metastatic lesions. Motor function was assessed right before the start of radiotherapy, as well as at one month, three months and six months after radiotherapy was completed. To categorize motor function we used a 5-point scale: Grade 0: normal strength; Grade 1: ambulatory without aid, Grade 2: ambulatory with aid, Grade 3: not ambulatory, Grade 4: paraplegia [10]. If the patient’s motor function was rated as improved or deteriorated, an alteration of at least one point on the 5-point scale must have had occurred. Patients who presented with complete paraplegia and did not approve were rated as deteriorated.

The following nine potential prognostic factors were investigated with respect to post-radiotherapy motor function, local control of MSCC, and survival: age (≤65 *vs.* >65 years), gender, Eastern Cooperative Oncology Group performance score (ECOG-PS 2 *vs.* 3–4), number of involved vertebrae (1–2 *vs.* 3), pre-radiotherapy ambulatory status (not ambulatory *vs.* ambulatory), other bone metastases at the time of radiotherapy (no *vs.* yes), visceral metastases at the time of radiotherapy (no *vs.* yes), time developing motor deficits before radiotherapy (1–7 *vs.* >7 days), and the radiotherapy schedule (short-course radiotherapy with 1x8 Gy or 5x4 Gy in 1 week *vs.* longer-course radiotherapy with 10x3 Gy in 2 weeks, 15x2.5 Gy in 3 weeks, or 20x2 Gy in 4 weeks).

The nine potential prognostic factors for functional outcome were included in a multivariate analysis performed with the ordered-logit model, because these data were ordinal (−1 = deterioration, 0 = no change, 1 = improvement of motor function). Local control was defined as no recurrence or progression of MSCC in the irradiated spinal region. The diagnosis of an in-field recurrence of MSCC was confirmed by computed tomography or magnet resonance imaging. Local control and survival rates were calculated with the Kaplan-Meier-method [11]. The differences between the Kaplan-Meier curves were calculated with the log-rank test. The prognostic factors found to be significant (p < 0.05) in the univariate analysis were included in a multivariate analysis, performed with the Cox proportion hazards model. Patients were followed until death or for median 7.5 months (range: 6–20 months) in those alive at the last follow-up visit.

## Abbreviations

ECOG, Eastern cooperative oncology group; ECOG-PS, Eastern cooperative oncology group performance score; Gy, Gray; MeV, Mega electron volts; MSCC, Metastatic spinal cord compression; RT, Radiotherapy.

## Competing interests

The authors declare that they have no competing interests.

## Authors’ contributions

SD, SES and DR participated in the design of the study. VR and SES performed the statistical analyses. SD, SH, AB and DR provided study material. All authors were involved in manuscript writing; they read and approved the final manuscript.

## Pre-publication history

The pre-publication history for this paper can be accessed here:

http://www.biomedcentral.com/1471-2407/12/261/prepub
